# McLeod syndrome with a novel *XK* frameshift mutation: A case report

**DOI:** 10.1097/MD.0000000000028996

**Published:** 2022-03-11

**Authors:** Shilin Xia, Xinrui Yu, Fan Song, Bo Sun, Ying Wang

**Affiliations:** a Clinical Laboratory of Integrative Medicine, The First Affiliated Hospital of Dalian Medical University, Dalian, China; b Department of Neurology, The First Affiliated Hospital of Dalian Medical University, Dalian, China; c Department of Radiology, The First Affiliated Hospital of Dalian Medical University, Dalian, China.

**Keywords:** chorea, frameshift mutation, McLeod syndrome, neuroacanthocytosis, *XK* gene

## Abstract

**Rationale:**

McLeod syndrome (MLS) is a rare X-linked neurohematologic disorder caused by loss-of-function mutations in the XK gene. However, variations in the XK gene remain to be elucidated. Here, we report the clinical phenotype and genetic features of a patient with MLS caused by a novel frameshift mutation in the XK gene.

**Patient concerns:**

A 44-year-old man presented with chorea, cognitive impairment, mental disorders, and seizures accompanied by peripheral neuropathy, hyperCKemia, and acanthocytosis. The proband's mother had a mild chorea. One older brother who died 10 years ago without a confirmed diagnosis showed symptoms of both chorea and mental disorders, while the other brother also developed mild chorea.

**Diagnosis:**

The patient was diagnosed with MLS based on the family history, clinical manifestations, and accessory examinations. Whole-exome sequencing studies revealed a novel frameshift mutation resulting from a nucleotide variation in exon 2 (452delA) that leads to an amino acid residue conversion from Gln to Arg and early termination of the XK protein (Gln151ArgfsTer2). The patient and one of his older brothers were hemizygotes, and his mother was heterozygous.

**Interventions:**

The patient was treated with haloperidol to control chorea and levetiracetam to control seizures.

**Outcomes:**

Six months after treatment, the proband was seizure-free, but showed little improvement in chorea and cognitive dysfunction.

**Lesson:**

We describe a family with MLS caused by a novel frameshift mutation in the XK gene. The causes of the mild clinical presentation in the proband's mother require further investigation.

## 1 Introduction

McLeod syndrome (MLS) is part of the spectrum of neuroacanthocytosis syndrome that affects the peripheral and central nervous systems, erythrocytes, and internal organs. It is defined as a rare X-linked disorder; however, variations in the XK gene await elucidation.^[1–3]^ In this report, we present the clinical phenotype and genetic features of a family with MLS caused by a novel frameshift mutation in *XK*.

## 2 Case presentation

**Figure 1:**
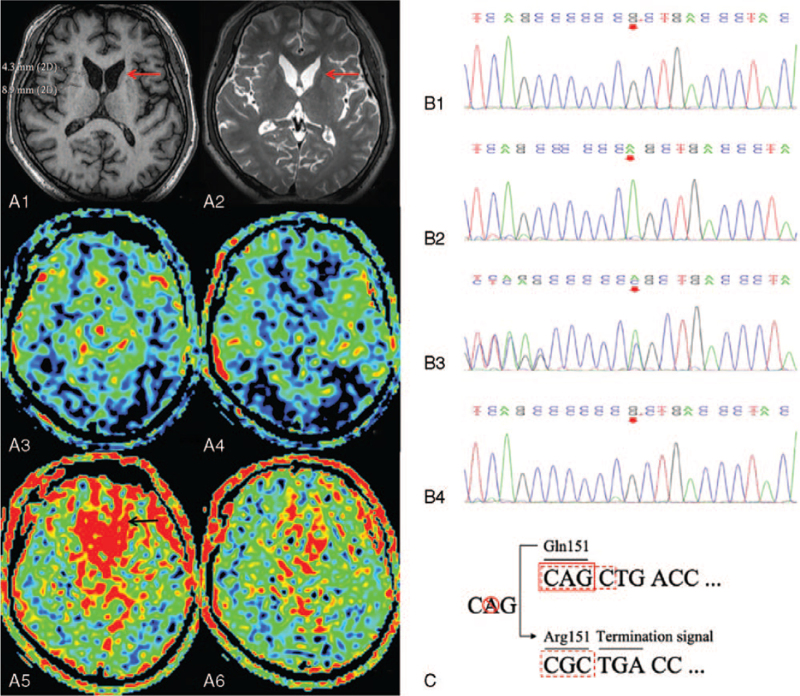
(A) The brain MRI of the patient with McLeod syndrome. (A1–A2) Axial view of T1 weighed image (A1) and T2 weighed image (A2) of the patient showed significant atrophy of bilateral caudate nuclei (red arrows) and mild atrophy of bilateral putamen. (A3–A6) Arterial spin labeling (ASL) images revealed a reduction in total cerebral perfusion (A3–A4) and an increase of the delayed frontobasal perfusion (A5–A6, see black arrow in A5). (B) Sequence analysis of the patient with McLeod syndrome and his family. (B1) Proband: a frameshift mutation in the *XK* gene. (B2) Father: no mutation in the *XK* gene. (B3) Mother: heterozygote of the *XK* gene. (B4) Brother: a frameshift mutation in the *XK* gene. (C) The frameshift mutation resulted in an amino acid sequence change from Gln to Arg-termination signal. MRI = magnetic resonance imaging.

A 44-year-old male was admitted to our hospital in January 2019, with “involuntary choreatic movements for more than 10 years, memory loss and emotional instability for 3 years, paroxysmal consciousness loss with convulsions for 1.5 years” as chief complaints. He developed involuntary chorea in the extremities approximately 10 years ago, which progressed gradually to his mouth, tongue, face, neck, and shoulders, accompanied by abnormal postures and unstable walking. Movement disorders were followed by memory loss, emotional instability, and childish behaviors approximately 7 years later. One and a half years ago, he began to experience paroxysmal consciousness loss with convulsions lasting for several minutes without an obvious aura that happened 1 to 2 times per month. No medical history has been reported to date. A family history indicated that the proband's mother had mild chorea. One elder brother, who died 10 years ago without a confirmed diagnosis, experienced chorea and mental disorders. The other elder brother also developed mild chorea; however, his sister, father, and son were all normal. The family pedigree is shown in Figure S1, Supplemental Digital Content. Neurological examination revealed that the patient exhibited slurred speech and irritation with childish behavior. Memory, orientation, and calculations were within the normal range. Choreatic movements were observed in the extremities, mouth, face, neck, and shoulder. An unstable posture and an awkward gait were observed. The muscle power of the extremities was normal, the muscle tone was reduced, and the tendon reflexes of all 4 limbs were reduced. Brain magnetic resonance imaging (MRI) revealed significant atrophy of the bilateral caudate nuclei and mild atrophy of the bilateral putamen. Arterial spin labeling revealed a reduction in total cerebral perfusion and an increase in delayed frontobasal perfusion (Fig. 1A). Electroencephalography showed slowing of the background activity and intermittent rhythmic delta activity in all electrode recordings. Electromyography and nerve conduction studies showed neurogenic damage at the cervical, thoracic, and lumbar segments, with sensorimotor axonal damage at the cervical segment and motor axonal damage at the thoracic and lumbar segments. The patient scored 21 points on the Mini-Mental State Examination, with impaired memory performance, particularly naming and writing ability. The Hamilton Anxiety Scale showed 4 points without anxiety performance. The Hamilton Depression Scale score was 9 points, indicating mild depression. The electrocardiogram was normal, and heart Doppler ultrasound showed mild mitral regurgitation. A peripheral blood smear revealed acanthocytes (3% in total). The level of creatine kinase (CK) was elevated to 825 U/L (38–174) and creatine kinase-MB was increased to 5.47 μg/L (0–5.00). Whole exon sequencing studies (Fig. 1B–C) revealed a novel frameshift mutation caused by a nucleotide variation in exon 2 (452delA) in the XK gene. This deletion of nucleotide 452 A in the coding region leads to an amino acid residue conversion from Gln to Arg and early termination of the XK protein (Gln151ArgfsTer2). The proband and one of his older brothers were hemizygotes, and his mother was heterozygous. This variant is not polymorphic and occurs at a very low frequency in the population (reference database: 1000Genomes, dbSNP). Based on the family history, clinical manifestations, and accessory examinations of the proband, MLS was diagnosed. He was treated with haloperidol to control the chorea and levetiracetam to control seizures. Six months after treatment, the proband was seizure-free, but little improvement was observed in chorea and cognitive dysfunction. The Mini-Mental State Examination scores remained unchanged after treatment.

## 3 Discussion

MLS is an X-linked disease caused by the loss or dysfunction of the XK protein due to loss-of-function mutations in the *XK* gene on the X chromosome.^[1–3]^ Based on exon sequencing analysis, we found a previously unreported frameshift mutation in exon 2 (c.452delA), resulting in amino acid residue conversion from Gln151 to Arg151. Consequently, the next triplet of bases is turned into a terminator. Its neighboring locus 451 was reported to have a base-duplicated mutation^[4]^; hence, further studies are needed to determine the mutational landscape of this region. It has been established that there are 39 types of *XK* gene mutations that can cause hematologic phenotypes and MLS,^[1–3,5]^ among which there are 2 frameshift mutations including loci 195-198 CCGC deletion and loci 640-645 TGGAGG deletion.^[1]^ Both were series base deletions. In this report, a frameshift mutation was caused by a single-nucleotide deletion, which is notable as a new frameshift variation in MLS. Understanding how this mutation contributes to pathogenicity in MLS may enable its use as a prognostic factor or therapeutic target.

Interestingly, the proband's mother, with a heterozygous mutation, who should theoretically be normal, had mild chorea. The negative result of the X-chromosome inactivation analysis excluded her symptoms caused by X-chromosome inactivation. To date, there has been only 1 female case of confirmed severe MLS, which was a heterozygote with a single nucleotide deletion (268delT terminating at AA129) in exon 2 of the *XK* gene. In this case, *XK* inactivation resulting from skewed lyonization contributes to severe MLS. The patient developed seizures in her fifties, with gradual progression of neurological symptoms, including chorea and cognitive impairment, before she died at 60 years of age. Both her sons had MLS. Her sister and niece developed mild neurological symptoms, including chorea in the lower extremities and ankle areflexia. The skewing of lyonization ranges from slightly skewed in the sister to completely normal in the niece.^[1,6]^ Age-related skewing of lyonization has been reported in female patients with progressive X-linked diseases,^[7]^ which could explain the difference in skew between affected women. Above all, the symptoms of the proband's mother in our report might be similar to those in the above pedigree and related to age-related skewing of lionization.

The key neurological manifestation of MLS is chorea, which develops in 70% of patients.^[5]^ In addition, parkinsonism, mental disorders, cognitive impairment, seizures, and neuromuscular involvement are all common.^[1–3,5,8,9]^ Symptoms in other systems include acanthocytosis, hemolytic anemia, hepatosplenomegaly, and cardiac damage.^[1–3,5,8,10]^ The proband presented with chorea as an initial symptom, followed by cognitive impairment, mental disorders, and seizures, accompanied by peripheral nerve and muscle involvement and acanthocytosis. One of his older brothers experienced chorea together with mental disorders 10 years ago and died without a confirmed diagnosis, while the other older brother also developed mild chorea. The clinical features of this family are typical, but the age of onset and clinical phenotype of the same family vary, which is consistent with previous studies.^[11]^ More than 60% of patients with MLS have cardiac involvement, including dilated cardiomyopathy and arrhythmia.^[10]^ In our case, the proband showed no clinical cardiac symptoms; however, an elevated level of creatine kinase-MB was observed, and further follow-up is needed to rule out the possibility of cardiac damage.

Brain MRI of the proband showed significant atrophy of the bilateral caudate nuclei and mild atrophy of the bilateral putamen, indicating an impairment of the striatum, which is consistent with previous studies,^[12]^ which revealed a reduction in global cerebral perfusion, which was consistent with whole brain symptoms.^[13]^ However, the reason for increased delayed frontobasal perfusion is unclear and requires further investigation. Previous studies have shown atrophy and low metabolism in the striatum of patients with MLS using MRI and positron emission tomography, which became more aggravated with disease progression, indicating that imaging is of great value in assessing disease progression.^[12,13]^ Our findings were consistent with a recent report indicating that in addition to the striatum, patients with MLS also showed low metabolism in the frontotemporal region. These findings could explain the psychiatric symptoms of the patient^[3]^ and further illustrate more extensive brain dysfunction.

Currently, the treatment for MLS focuses on symptom amelioration.^[1,14]^ Dopamine depletory drugs such as tetrabenazine and reserpine are administered to improve chorea. Haloperidol, a dopamine receptor antagonist that may cause bradykinesia and muscle rigidity, and thus not recommended as a first-choice, can be used at a later stage. The treatment of psychiatric symptoms, cardiac abnormalities, and seizures is based on clinical findings and patient status.^[1,3,14]^ Regardless, long-term and continuous multidisciplinary support, including genetic counseling, is needed for patients and their families. When patients with MLS need a blood transfusion, the care and support of specialist transfusion institutions are required because of the rare blood group phenotype characterized by the absence of Kx antigen and reduced expression of Kell antigens. In such cases, immunohematology and molecular biology tests are demanding, and inter-institutional or international collaboration for the provision of compatible units is usually required because of the rarity of compatible blood products. In any case of transfusion, Kx+ transfusions should be avoided in both male and female patients with a McLeod phenotype. Autologous banked donations are the most suitable transfusion practices, if feasible.^[1]^

## 4 Conclusion

Overall, we describe a rare family with MLS caused by a novel frameshift mutation in *XK*. The causes of the mild clinical presentation in the proband's mother are unclear and require further investigation.

## Acknowledgments

We are grateful to the patient and his family who participated in this study.

## Author contributions

Ying Wang is the corresponding author who contributed to study design and manuscript writing. Bo Sun contributed to the study design and imaging analysis. Shilin Xia and Xinrui Yu contributed equally to the clinical data collection, analysis, and manuscript writing. Fan Song collected clinical data and revised the manuscript. Shilin Xia and Xinrui Yu contributed equally to the clinical data collection, analysis and manuscript writing. Ying Wang and Bo Sun contributed equally to the study design. All authors have read and approved the final manuscript.

**Conceptualization:** Xinrui Yu, Bo Sun, Ying Wang.

**Data curation:** Shilin Xia, Fan Song.

**Formal analysis:** Xinrui Yu.

**Funding acquisition:** Ying Wang.

**Investigation:** Shilin Xia, Fan Song.

**Methodology:** Shilin Xia, Fan Song, Bo Sun.

**Supervision:** Ying Wang.

**Writing – original draft:** Shilin Xia, Fan Song.

**Writing – review & editing:** Xinrui Yu, Bo Sun, Ying Wang.

## Supplementary Material

Supplemental Digital Content
