# Supplementary material for: McLeod syndrome with a novel XK frameshift mutation: A case report
Source: Medicine (Baltimore). 2022 Mar 11;101(10):e28996. doi: 10.1097/MD.0000000000028996 (PMC8913091; doi:10.1097/MD.0000000000028996)
Supplement: Supplemental Digital Content [file medi-101-e28996-s001.doc]

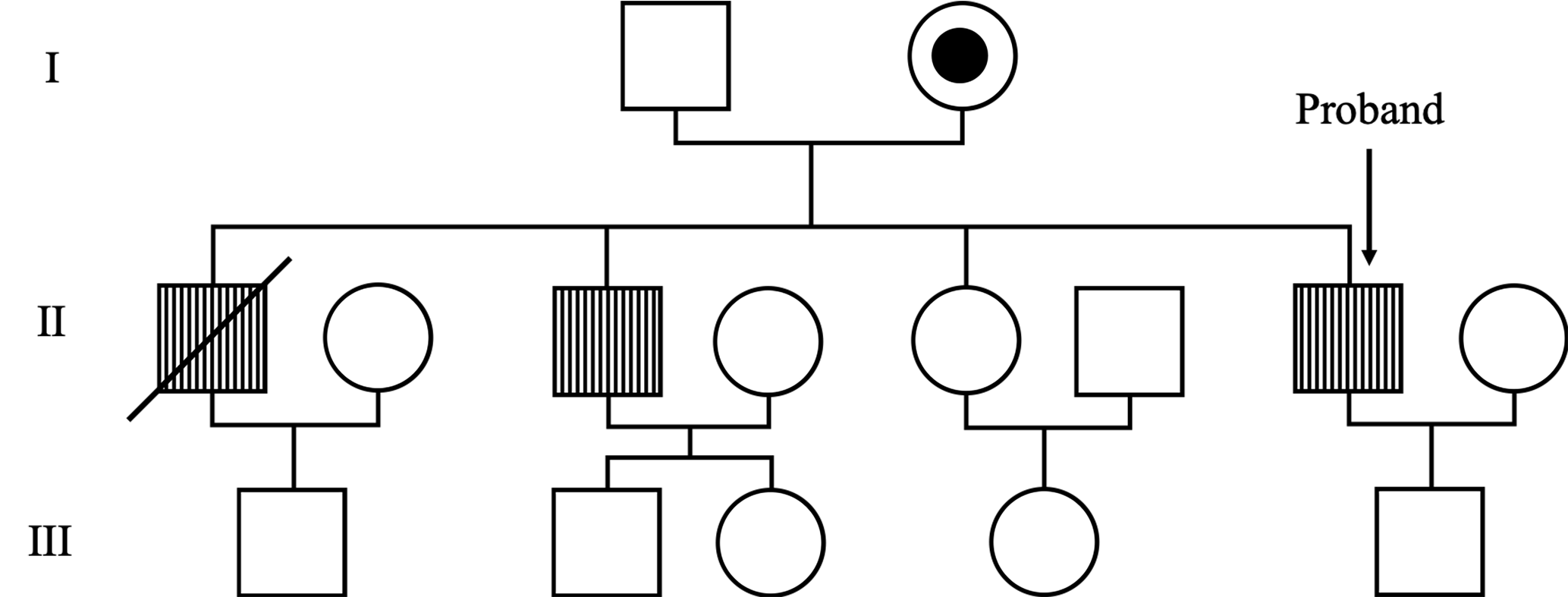


**Supplementary Figure 1.** Family pedigree of the patient of McLeod syndrome.


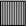
⦿: Female heterozygote. ○: Health female. □: Health male. : Male hemizygote.


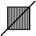
 : Death.
